# Incidence and Prevalence of Multisystem Inflammatory Syndrome in Children (MIS-C) in Southern Italy †

**DOI:** 10.3390/children10050766

**Published:** 2023-04-23

**Authors:** Francesco La Torre, Maria Pia Elicio, Viviana Anna Monno, Maria Chironna, Fulvio Moramarco, Angelo Campanozzi, Adele Civino, Valerio Cecinati, Ugo Vairo, Mario Giordano, Leonardo Milella, Daniela Loconsole, Fabio Cardinale

**Affiliations:** 1 Department of Pediatrics, Giovanni XXIII Pediatric Hospital, University of Bari, 70126 Bari, Italy; 2 Department of Interdisciplinary Medicine, Hygiene Section, University of Bari, 70123 Bari, Italy; 3 Department of Pediatrics, Antonio Perrino Hospital, 72100 Brindisi, Italy; 4 Department of Medical and Surgical Sciences, Pediatric Section, University of Foggia, 71122 Foggia, Italy; 5 Division of Pediatric Rheumatology and Immunology, Vito Fazzi Hospital, 73100 Lecce, Italy; 6 Department of Pediatrics, SS Annunziata Hospital, 74100 Taranto, Italy; 7 Division of Pediatric Cardiology, Giovanni XXIII Pediatric Hospital, 70126 Bari, Italy; 8 Division of Pediatric Nephrology and Dialysis, Giovanni XXIII Pediatric Hospital, 70126 Bari, Italy; 9 Division of Intensive Care, Giovanni XXIII Pediatric Hospital, 70126 Bari, Italy

**Keywords:** children, MIS-C, COVID-19, incidence, epidemiology

## Abstract

Multisystem inflammatory syndrome in children (MIS-C) is a pediatric hyperinflammatory syndrome related to severe acute respiratory syndrome coronavirus 2 (SARS-CoV-2) infection whose epidemiology is not very well known at present. The objective of the study was to better understand the incidence of MIS-C in the Apulia region in southern Italy. Our primary goal was to estimate the incidence of newly identified cases of MIS-C in children aged 0–18 years, during a period of six months, encompassing the second pandemic wave. We also analyzed the characteristics of our cohort in terms of clinical features, treatment, and outcomes. The cumulative incidence of MIS-C was 3.27 per 100,000 residents between 0 and 18 years of age. In our cohort, gastrointestinal, mucocutaneous, and cardiac involvement were the most common clinical features. With our step-up approach to therapy, no patients required intensive care unit (ICU) admission and no cardiac sequelae after 6 months of onset were found in echocardiograms. Conclusion: Our epidemiological study of MIS-C in southern Italy showed unexpectedly overlapping figures with other US studies.

## 1. Introduction 

Incidence of the novel virus called SARS-CoV-2 has rapidly increased around the world since late 2019. In February 2020, the World Health Organization (WHO) called the disease resulting from its infection coronavirus disease 2019 (COVID-19) [1]. Most children and adolescents with COVID-19 experience an asymptomatic or mild disease course [2]. Nonetheless, in late April 2020, soon after the first wave of SARS-CoV-2 infections in Italy, there was an unusual peak in children presenting with a severe systemic inflammatory disease resembling Kawasaki Disease (KD) who required hospitalization [2]. The first cases were published soon after by Verdoni [3]. In the following weeks, a Kawasaki-like disease began to be reported in some pediatric patients with a history of COVID-19 worldwide [4,5,6]. This condition is now called multisystem inflammatory syndrome in children (MIS-C) [1,2,6,7,8,9]. It is characterized by hyperinflammatory and multiple organ involvement and usually occurs 2–6 weeks after the acute phase of SARS-CoV-2 infection [1,2,6,7,8,9]. 

MIS-C has some overlapping findings with KD, with a range of clinical features including mucocutaneous, pulmonary, gastrointestinal, neurological, and cardiovascular symptoms. Unlike KD, however, MIS-C has been suggested to predominantly affect adolescents and children older than five years of age and to be associated more frequently with gastrointestinal involvement and myocarditis [1,2,6,7,8,9,10]. In fact, gastrointestinal symptoms such as abdominal pain, vomiting, and diarrhea are the predominant clinical manifestation in MIS-C patients and were found in 70–90% of cases [6,9,10,11,12,13,14,15,16,17,18]. Cardiac involvement was found in 35–80% of patients, and the combined prevalence of myocarditis or pericarditis was 34.3% [6,9,19]. The incidence of coronary artery dilatation varied between 10% and 48% in different reports [15,20,21]. Mucocutaneous signs were similar to those observed in KD [9,14,16,22,23,24]. The neurological manifestations, from mild symptoms such as headache or transient impairment of consciousness to severe manifestations such as seizures or aseptic meningoencephalitis, have also been described with a variable incidence from 11% to 67% [25,26]. Many patients presented hypotension and shock that frequently required intensive care. [6,9,11,12,13,14,15,16,17]. Another severe complication in MIS-C patients was represented by macrophage activation syndrome (MAS). It was present in 15–25% [16,27,28], according to the international diagnostic criteria for MAS for rheumatic disease [27]. 

Due to the overlap with commonly observed features of KD, patients with MIS-C are currently treated based on KD therapy protocols. The American College of Rheumatology (ACR) has provided clinical practice guidelines for the laboratory workup and management of patients with suspected MIS-C [29]. High-dose intravenous immunoglobulin (IVIG) at 2 g/kg and intravenous corticosteroids at 2 mg/kg/day, in divided doses, have been recommended as the starting treatment. Steroids have been shown to have a favorable effect in MIS-C patients compared to IVIG monotherapy [30,31]. Patients were also started on low-dose aspirin (3–5 mg/kg, max 100 mg daily). For MIS-C with significant left ventricular dysfunction evidenced by Ejection Fraction (EF) < 55%, anticoagulation with low molecular weight heparin (LMWH) is usually considered [29,32]. Patients with severe clinical presentation (significant cardiovascular involvement, evidence of MAS) have been treated with high-dose methylprednisolone (30 mg/kg/day, max 1000 mg per dose, for three consecutive days) followed by sparing dose (2 mg/kg/day in divided doses) [10]. The use of anakinra, an IL-1 receptor antagonist, has been indicated by ACR in cases of refractory MIS-C despite IVIG and steroids, or in case of severe myocarditis or MAS [29,32]. Many papers have shown the efficacy of anakinra in MIS-C patients [33,34,35]. Nevertheless, due to the severe inflammation, higher doses of intravenous anakinra (>4 mg/kg/day, up to 10 mg/kg/day, in divided doses) are usually considered, particularly when there is evidence of MAS or a severe systemic involvement [29,33,34]. A significant proportion of patients required intensive care (31–85%), and some of them needed mechanical ventilation and extracorporeal membrane oxygenation [9,14,16,36]. Patients can deteriorate quickly, requiring cardiac or respiratory support, with poor prognosis related to the involvement of the cardiovascular system accompanied by systemic inflammation, severe cardiac disease, shock, disease of the coronary arteries, and/or MAS [37]. A mortality rate of 1–5% was reported [36,37].

Very few studies have analyzed the incidence of MIS-C to date. Most of these studies come from the United States (US) and largely reflect North America’s racial and environmental backgrounds [9,38,39]. The median age in these studies is 7.3–10.8 years old, and 55% of patients were male [40,41]. Many of the studies reported a higher incidence of MIS-C in Hispanic, African, or Latino–American subjects [9,38,39,40,41]. About 23% of patients had comorbidities, and obesity/overweight was the most frequent, followed by cardiac and respiratory features [41]. During the period between 1 March and 10 May 2020, an incidence of two cases per 100,000 subjects under 21 years old was reported in the state of New York [9]. In another survey, conducted by Lee in New York City, the incidence of MIS-C was 11.4 per 100,000 patients under 20 years of age during the period between 1 March and 30 June 2020 [38]. In a further study published by Payne in a cohort of 248 patients with MIS-C in the period between 1 April and 30 June 2020, an incidence of 5.1 per 1,000,000 person/month was estimated. In the same paper, a prevalence of 316 cases per 1,000,000 SARS-CoV-2 infections in persons younger than 21 years was calculated [39]. In another study, conducted in Cape Town, South Africa, a prevalence of 22 cases of MIS-C per 100,000 SARS-CoV-2 infections in children younger than 14 years was found [42]. Since the first descriptions, the incidence of MIS-C has been decreasing over time [43]. Data on the incidence of MIS-C in Europe are missing. Epidemiological data are only present in the United Kingdom (UK) and only include patients under 15 years of age.

The purpose of this study was to estimate the Incidence of MIS-C in Apulia, a geographical region located in southern Italy with a population of about four million, evaluating newly identified cases of MIS-C in children 0–18 years of age during the second wave of the pandemic. We also calculated the prevalence of children with previous SARS-CoV-2 infection who developed MIS-C and the cumulative incidence per 100,000 residents, distributed according to four age groups, ≤5, 6–10, 11–15, 16–18 years, and the incidence rate of MIS-C in terms of cases/month/year. We also described the characteristics of our MIS-C cohort in terms of clinical features, treatment, and outcome.

## 2. Materials and Methods

### 2.1. Study Design

An observatory network for all the pediatric departments in Apulia was established in October 2020. Data from all cases of MIS-C observed in patients between 0 and 18 years, hospitalized in all regional pediatric departments, over a period of six months spanning from 1 November 2020 to 30 April 2021, were collected. All children diagnosed as having MIS-C in accordance with the criteria developed by the World Health Organization (WHO) were included in the study (Table 1) [44].

### 2.2. Statistical Analysis

The collected data were analyzed using STATA MP17 software. Continuous variables were expressed as mean ± standard deviation and range, categorical variables as proportions. The data source was the “Infections Regional Informative System” (IRIS)-Apulia. This is a very rigorous regional informative system that collects all positive cases of SARS-CoV-2. Cases are inserted by all public health system workers who perform and test positive for COVID-19. The number of residents aged 0–18 years in Apulia relating to the 2020–2021 data was obtained from the Italian National Statistics Institute (ISTAT) registry. The prevalence was calculated by dividing the number of MIS-C patients by the total individuals 0–18 years at risk of the disease (confirmed SARS-CoV-2 infection).

## 3. Results

During the reporting period, 22 new cases of MIS-C were admitted to five community pediatric departments in Apulia (Bari, Brindisi, Foggia, Lecce, and Taranto). These are reference hospitals for the region, which generally collect the most complex cases for all pediatric pathologies. Therefore, the diagnoses were made in these departments by pediatricians with great experience in the management of patients with Kawasaki disease and other systemic vasculitis. All the cases were Caucasians and there was a higher prevalence in males (14 boys and 8 girls–M/F ratio: 1.7). The median age was 7.54 years (IQR 2.60–12.80). Comorbidities such as obesity/overweight were present in 5 out 22 of patients (23%). Nasopharyngeal swabs and serological tests for SARS-CoV-2 gave positive results during hospitalization in 1/22 and 22/22 of patients, respectively. The cumulative incidence of MIS-C was 3.27 per 100,000 residents between 0 and 18 years of age (Table 2). 

**Figure 1:**
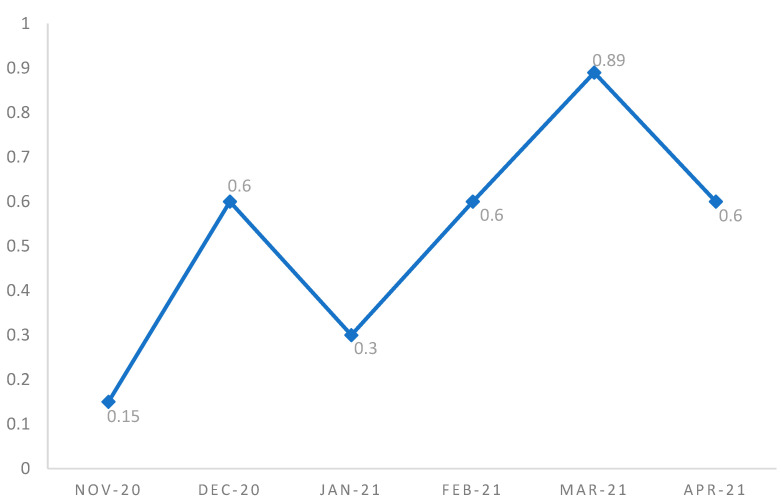
Incidence rate per month (×100 000 residents 0–18 years) of MIS-C.

The number of children 0–18 years with SARS-CoV-2 infection confirmed by nasopharyngeal swab during the six-month period was 29,585, and the prevalence of MIS-C among pediatric subjects with previous SARS-CoV-2 infection was 0.07%. We also evaluated the incidence according to four age groups: ≤5 years, 6–10 years, 11–15 years, and 16–18 years (Table 2). The most affected age group was 6–10 years, with a cumulative incidence of 5.67 per 100,000 residents aged 0–18 years. Finally, in our cohort, no cases of MIS-C between 16 and 18 years were recorded (Table 2). Most newly diagnosed cases of MIS-C followed the peak in SARS-CoV-2 infections after 2–6 weeks, and a notably greater number of cases was recorded in December 2020 and between late February and early April 2021, with a higher incidence rate per month, expressed in cases/month/year, being recorded in March 2021 (Figure 1). In particular, the incidence rate of MIS-C in the population between 0 and 18 years per 100,000 residents was 0.15 cases in November 2020, 0.6 cases in December 2020, 0.3 cases in January 2021, 0.6 cases in February 2021, 0.89 cases in March 2021, and 0.6 cases in April 2021 (Figure 1).

Gastrointestinal involvement with abdominal pains was the most frequent organ involvement (19/22, 86%). Of these patients, 12/19 had diarrhea, 4/19 had pancreatitis, 2/19 peritonitis, and 1/19 had paralytic ileus. In our cohort, abdominal imaging showed peritoneal effusion in 8 out 19 patients and, more frequently, wall-thickening of the intestinal tract, in 11 out 19 patients. Cardiovascular involvement was present in 18 out 22 patients (81.8%). Signs of myocarditis with an increase in troponin and brain natriuretic peptide (pro-BNP) were present in half of patients (11/22). A coronary artery ectasia was observedin four patients. Pericarditis and endocarditis were present in 8 out 22 and 4 out 22 patients, respectively. The involvement of other organs, in order of frequency, was as follows: conjunctivitis (18/22), mucositis (18/22), rash (17/22), neurological (7/22), respiratory (5/22), capillary leak syndrome (5/22), macrophage activation syndrome (MAS) (5/22) (Table 3). After characterizing the laboratory findings, lymphopenia (<1000 mmc) and thrombocytopenia (<110.000 mmc) were found to be present in 13 out 22 and 8 out 22 patients, respectively. Hyperferritinemia(>500 ng/mL) and hypoalbuminemia (25 g/L) were also present in 11 out 22 and 5 out 22 patients, respectively (Table 3). All patients received one dose of 2 g/kg (max 80 gr) of intravenous immunoglobulin (IVIG), but only two patients (9%) had a rapid and satisfactory response. The remaining 20 patients were also treated with steroids. Six nonresponder IVIG patients (27%) with noncomplicated organ involvement started with low-dose intravenous (IV) methylprednisolone (2 mg/kg/day) and then tapered using oral prednisone. Fourteen patients (63%) showing the presence of myocarditis, aseptic meningoencephalitis, shock, or MAS, received high-dose methylprednisolone at a dose of 30 mg/kg/day, with a maximum (max) 1000 mg per dose for three consecutive days, and were then tapered with low-dose IV methylprednisolone and oral prednisone. Of this latter group, in five patients (23%) with severe cardiac involvement (EF < 55%) and/or MAS, treatment with endovenous anakinra at a dose of 2 mg/kg (max 100 mg) four times/day was also started. In these patients, after achieving control of the disease with normalization of CRP, ferritin, and signs of myocarditis, anakinra was reduced by one dose every 3 days until discontinuation. All patients were treated with oral aspirin (ASA) at 5 mg/kg (max 100 mg/day). Subcutaneous LMWH with enoxaparin at 100 unit/Kg/day was started for thromboprophylaxis before ASA in patients with severe organ involvement or elevated D-dimer levels (13/22) (Table 3). All children completely recovered with this step-up approach to therapy and no patients required pediatric intensive care unit (PICU) admission. Upon discharge, 5 out 22 (23%) presented with residual cardiac involvement (one with mild right coronary ectasia, two with mitral insufficiency, one with aortic insufficiency, and one with mild pericardial effusion). In the follow-up visit, at 8 weeks from onset, all residual cardiac abnormalities were resolved, and ASA therapy was stopped for all patients. No cardiac sequelae were found in echocardiogram 6 months after onset (Table 3).

## 4. Discussion

### 4.1. Epidemiological Data

**Figure 2:**
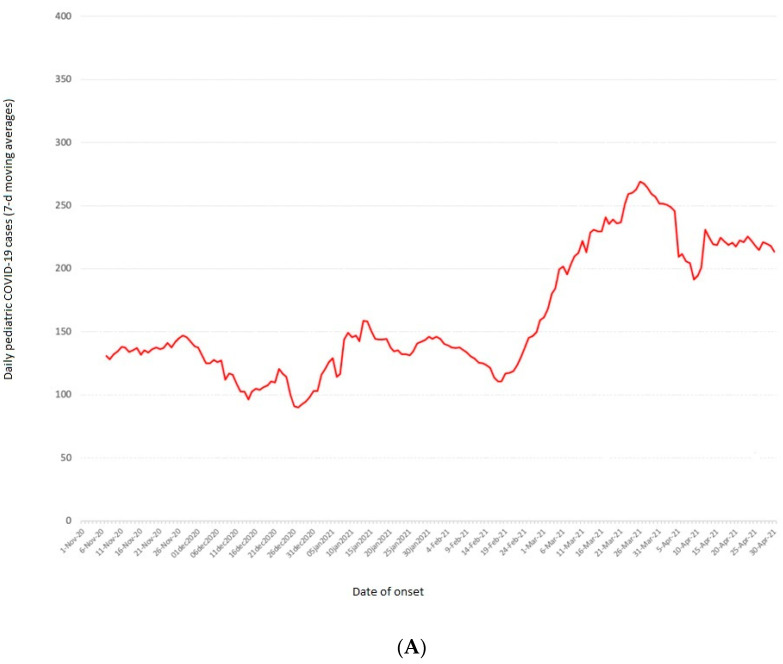

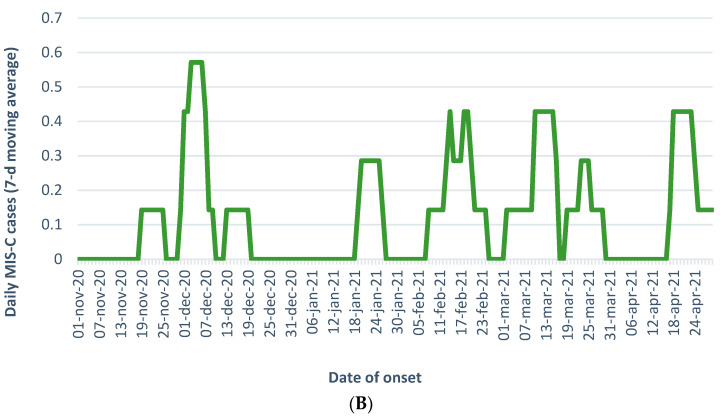
Curves of moving average of COVID-19 cases after 7 days (red line) in Apulian population 0–18 years (**A**) and curves of moving average of MIS-C cases after 7 days (green line) in Apulian population 0–18 years (**B**).

Our study showed unexpectedly overlapping figures with some studies conducted in the US. In fact, our data displayed a very similar trend of MIS-C incidence in Italy to that published by Dufort in the US, which also showed a higher cumulative incidence in the age group 6–10 years [9]. In our cohort, we could not evaluate the role of racial factors because all the children enrolled were Caucasian. Regarding the sex ratio, according to previously published data, a higher prevalence was also found in males (63.63%). The median age of our cohort was 7.54 years, in the lower end of the reported range (7.3–10.8 years) for MIS-C [4,19,37], although this was older compared to the published range for Kawasaki Disease, which is mainly shown to affect children aged less than 5 years [1,2]. Moreover, in our study, about 1 in every 1400 children who had contracted COVID-19 was at risk of developing MIS-C. In fact, the number of children 0–18 years with confirmed SARS-CoV-2 infection during the six months considered was 29,585, and the prevalence of MIS-C among pediatric subjects with previous SARS-CoV-2 infection was 0.07%, with a total of 74 cases per 100,000 younger than 18 years. However, due to the frequent presence of asymptomatic infections in childhood, these data are certainly overestimated. Furthermore, as already widely reported in the literature, in our cohort, MIS-C also occurred 2–6 weeks after COVID-19 infection and its recrudescence followed the peak in positivity for SARS-CoV-2, with a peculiar concordance with both epidemic curves (Figure 2) [39].

Figure 2 illustrates the epicurve of the COVID-19 cases (Figure 2A) using 7 day moving averages, contrasted with that of the MIS-C cases (Figure 2B) in the Apulian population 0–18 years. Figure 2B shows three peaks for MIS-C in December 2020, late February–early March 2021, and April 2021. The first two peaks followed those due to the COVID-19 pandemic by 2–6 weeks. The third peak seems to follow the peak in the COVID-19 pandemic in Apulia. As illustrated in Figure 2B, the first MIS-C peak (December 2020) was primarily due to the first phase of the second pandemic wave, which started in our region in late October 2020 and continued in November 2020 (Figure 2A). Instead, the second MIS-C peak (late February–early March 2021, Figure 2B) was due to the rebound wave of COVID-19 cases that occurred after the Christmas period (Figure 2A). In contrast, the third MIS-C peak (April 2021, Figure 2B) followed the rise in the curve of COVID-19 cases in the Apulian population aged 0–18 years in March 2021 (Figure 2A).

### 4.2. Characteristics of MIS-Cohort (Clinical Features, Treatment, and Outcome)

The clinical features of MIS-C observed in our patients showed the similarities and differences between MIS-C and Kawasaki Disease outlined in the literature. Common clinical features in our cohort were conjunctivitis and mucocutaneous signs, similar to KD. Gastrointestinal and cardiac involvement with myocarditis were also frequent in our population and are known to be less common in KD [2,10,21]. In fact, gastrointestinal system with abdominal pains was the most frequent organ involved (87%), with a published prevalence of 70–90% [6,9,10,19]. More than half of patients (54%) had diarrhea, 18% had pancreatitis, 9% had peritonitis, and one patient (4,5%) had paralytic ileus. It is noteworthy that pancreatitis, confirmed by an evident increase in lipase (>1000 U/L), is not as rare an event as it is considered in the literature, because it was present in almost 1/5 of patients [18]. Wall-thickening of intestinal tract, as determined by ultrasound, was present in half of the patients. Cardiovascular involvement was evaluated in 82%. Signs of myocarditis were present in 50% of cases with an increase in pro-BNP (>1000 pg/mL, normal range 0–125) in all of these patients and, in 7 out 11, this was above 10,000 pg/mL. An increase in troponin (>500 pg/mL, normal range 0–70) was present in 4 out 11 patients. A reduction in left ventricular ejection fraction (LVEF) of 55–50% was present in 3 out 11 patients, but this was rapidly responsive to anakinra treatment in all cases. In fact, in our cohort, there was a similar prevalence of myocarditis compared to the published data [6,9,10,19], but the early combined approach adopted in these cases, with high doses of steroids and anakinra, reduced the percentage of patients with LVEF to below 55% and reduced the need for PICU admission [36,37]. The prevalence of coronary artery involvement was in line with other reports [17,22,23]. In fact, coronary artery ectasia was observed in 4 out 22 patients (18%) and, in all cases, the right coronary artery was involved. In our cohort, the prevalence of pericarditis (36%) and endocarditis (18%) was also similar to the other reports [19,22]. Coagulopathy, thrombocytopenia, and lymphopenia were more frequent in MIS-C than in KD [2,10,21]. Neurological involvement was present in 32%, as described in the literature (11–67%) [25,26]. All of these patients presented headache and, in 4 out 7, signs of aseptic meningoencephalitis with transient impairment of the consciousness and rigor nucalis were present. In these cases, there were also signs of myocarditis and an early combined approach was started, with high doses of steroids and anakinra treatment, resulting in a progressive improvement in the neurological picture up to regression. In our cohort, the prompt step-up treatment with IVIG, steroids, and anakinra depending on the organ involvement or response to therapy, contributed to the good outcomes in term of PICU admission and long-term cardiac sequelae. This is unlike KD, where steroids or anakinra are only used in refractory cases [2]. As such, the proportion of steroid use (86.3%) was higher in our cohort compared to the data reported (range 49–63%) [10,21]. The American College of Rheumatology (ACR) recommends a stepwise approach to the immunomodulatory treatment of MIS-C, with IVIG and moderate to high doses of glucocorticoids [29]. Observational studies have shown that starting therapy with IVIG plus corticosteroids was associated with a more favorable course of fever and less risk of heart dysfunction compared to IVIG alone, while their timely administration can prevent progression and the need for admission to intensive care [30,40,41]. The use of anakinra is recommended by ACR in cases of refractory MIS-C despite IVIG and steroids [29], but with our management strategy, the outcomes of our series were favorable, with no PICU admission cases (versus the reported range of 31–85%) [9,14,16,36], and no cardiac sequelae were seen with echocardiograms after 6 months from onset. In fact, even if, upon discharge, 5 out 22 (23%) patients presented with residual cardiac involvement (one mild right coronary ectasia, two mitral and one aortic regurgitation, and one mild pericardial effusion) in the follow-up visit 8 weeks after onset, all abnormalities were resolved. These data confirm that a prompt and aggressive treatment with a high dose of steroids and anakinra in severe MIS-C patients with myocarditis, meningoencephalitis, shock, or MAS can modify the outcome in these patients [45,46,47]. 

### 4.3. Strengths and Limitations of the Study

The strength of our study was the creation of a network among all the pediatric departments in a well-defined geographical region, which allowed us to catch all cases of MIS-C that occurred in the examined period. This makes our epidemiological data as accurate as possible and confirms the trends of the US data in Europe. One limit of our survey is the small sample size. It is also limited by being a retrospective study, although the data were prospectively inserted from our network. 

## 5. Conclusions

As far as we know, our work represents one of the first epidemiological studies of MIS-C in Europe, showing overlapping data with US studies. The cumulative incidence of 3.27 per 100,000 residents and prevalence of 74 cases per 100,000 pediatric subjects with previous SARS-CoV-2, between 0 and 18 years of age, were similar to those in other data described in the literature for the same period. Moreover, in our cohort, the prevalence of clinical features and laboratory findings are in line with the published data, showing a higher prevalence of gastrointestinal and cardiac involvement with myocarditis compared to Kawasaki disease. Early diagnosis and step-up treatment with IVIG, steroids, and anakinra depending on the organ involvement or response to therapy, contributed to the good outcomes achieved for our patients: no patient died, no patient was admitted to the PICU, and no cardiac sequelae were seen in echocardiograms taken 8 weeks from onset.

## Figures and Tables

**Table 1:** WHO criteria for diagnosis of MIS-C.

Organ Involvement	Inflammatory Markers	Evidence of SARS-CoV-2 Infection
**Mucocutaneous** (rash or mucocutaneous inflammation signs) or **Conjunctivitis** (bilateral nonpurulent)	Erythrocyte sedimentation rate (ESR)	Oronasal swab-PCR (RT-PCR), antigen test
**Hypotension** or **Shock**		
**Cardiac** (features of myocardial dysfunction, pericarditis, valvulitis, or coronary abnormalities, including ECHO findings or elevated Troponin/NT-proBNP)	C-reactive protein (CRP)	Serology
**Coagulopathy** (PT, PTT, elevated D-Dimers)	Procalcitonin	Contact with patients with COVID-19.
**Acute gastrointestinal symptoms** (diarrhea, vomiting, or abdominal pain)		

MIS-C diagnosis was made according to the presence of fever for 3 days or more, plus at least two of the organs involved, plus the elevation of inflammatory markers and evidence of COVID-19 infection (RT-PCR, antigen test or serology positive or possible contact with patients with COVID-19).

**Table 2:** Cumulative incidence of MIS-C by age group.

Age Group, y	Number of Cases	Cumulative Incidence (×100,000 residents 0–18 years)
≤5	9	5.02
6–10	10	5.67
11–15	3	1.56
16–18	0	0.00
0–18	22	3.27

**Table 3:** Characteristics of our MIS-C cohort in terms of clinical features, laboratory findings, treatment, outcome, and comorbidities.

Organ Involvement	n. Pts (Tot. 22)	%
Gastrointestinal	19	87
Cardiovascular	18	82
Conjunctivitis	18	82
Mucositis	18	82
Rash	17	77
Myocarditis	11	50
Pericarditis	8	36
Endocarditis	4	18
Neurological	7	32
Respiratory	5	23
MAS	5	23
**Laboratory findings**		
Lymphopenia (<1000 mmc)	13	59
Thrombocytopenia (<110.000 mmc)	8	36
Hyperferritinemia (>500 ng/mL)	5	23
Hypoalbuminemia (25 g/L)	11	50
**Treatment**		
IVIG 2 g/kg (max 80 gr)	22	100
Low-dose methylprednisolone (2 mg/kg/day)	6	27
High-dose methylprednisolone (30 mg/kg/day × 3 days)	14	64
Anakinra 2 mg/kg (max 100 mg) × 4 times/day	5	23
Aspirin 5 mg/kg (max 100 mg/day)	22	100
Enoxaparin	13	59
**Outcome**		
Upondischarge	5	23
After 8 weeks from onset	0	0
After 6 months from onset	0	0
**Comorbidity**		
Obesity/overweight	5	23

## Data Availability

The data analyzed during the current study are available from the corresponding author on reasonable request.
